# Flexible Polyolefin Elastomer/Paraffin Wax/Alumina/Graphene Nanoplatelets Phase Change Materials with Enhanced Thermal Conductivity and Mechanical Performance for Solar Conversion and Thermal Energy Storage Applications

**DOI:** 10.3390/polym16030362

**Published:** 2024-01-29

**Authors:** Jie Tian, Chouxuan Wang, Kaiyuan Wang, Rong Xue, Xinyue Liu, Qi Yang

**Affiliations:** 1 School of Civil Engineering and Architecture, Shaanxi University of Technology, Hanzhong 723099, China; 13366086793@163.com; 2 National and Local Engineering Laboratory for Slag Comprehensive Utilization and Environment Technology, School of Materials Science and Engineering, Shaanxi University of Technology, Hanzhong 723099, China; wangchouxuanxuan@163.com (C.W.); wangkaiyuan6@126.com (K.W.); 18582481950@163.com (R.X.); 1809179387@163.com (X.L.); 3 College of Polymer Science and Engineering, Sichuan University, Chengdu 610065, China

**Keywords:** phase change materials, injection moulding, solar energy storage, photo-thermal conversion efficiency

## Abstract

In this study, electrically insulating polyolefin elastomer (POE)-based phase change materials (PCMs) comprising alumina (Al_2_O_3_) and graphene nanoplatelets (GNPs) are prepared using a conventional injection moulding technique, which exhibits promising applications for solar energy storage due to the reduced interfacial thermal resistance, excellent stability, and proficient photo-thermal conversion efficiency. A synergistic interplay between Al_2_O_3_ and GNPs is observed, which facilitates the establishment of thermally conductive pathways within the POE/paraffin wax (POE/PW) matrix. The in-plane thermal conductivity of POE/PW/GNPs 5 wt%/Al_2_O_3_ 40 wt% composite reaches as high as 1.82 W m^−1^K^−1^, marking a remarkable increase of ≈269.5% when compared with that of its unfilled POE/PW counterpart. The composite exhibits exceptional heat dissipation capabilities, which is critical for thermal management applications in electronics. Moreover, POE/PW/GNPs/Al_2_O_3_ composites demonstrate outstanding electrical insulation, enhanced mechanical performance, and efficient solar energy conversion and transportation. Under 80 mW cm^−2^ NIR light irradiation, the temperature of the POE/PW/GNPs 5 wt%/Al_2_O_3_ 40 wt% composite reaches approximately 65 °C, a notable 20 °C improvement when compared with the POE/PW blend. The pragmatic and uncomplicated preparation method, coupled with the stellar performance of the composites, opens a promising avenue and broader possibility for developing flexible PCMs for solar conversion and thermal storage applications.

## 1. Introduction

As electronics and energy storage devices become more tightly packed, high-performance thermal interface materials (TIMs) are gaining attention [1,2,3]. Overheating leads to the decline of performance, reduced lifespan, and safety risks due to a sudden increase in internal heat flux. To ensure safety within a temperature range, it is vital to advance the development of effective materials and technologies for thermal management to ensure the prompt dissipation of accumulated heat. Moreover, given the pressing issues and concerns relating to the population growth, the environment, and energy challenges, the demand for eco-friendly energy has intensified [4,5,6].

Phase change materials (PCMs) are used to store energy and release excess heat during phase changes, making them a key component of thermal management and storage [4,7,8,9]. In general, PCMs mainly include organic PCMs and inorganic PCMs [10]. Compared with inorganic PCMs, organic PCMs have a low supercooling degree, high storage density, and no corrosivity, making them more widely used in industrial sectors [4]. Paraffin wax (PW), which is recognized for its versatility as an organic material, is highly valued for its capacity to adjust its phase change temperature, substantial latent heat capacity, and cost effectiveness [11,12,13]. These attributes make it applicable across various thermal management domains, such as solar energy, electronics, and power batteries. However, during practical use, PW-based phase change composites (PWPCMs) face substantial challenges, exhibiting an inherent low thermal conductivity, potential leakage, inefficient light-to-heat conversion, and lack of flexibility, which decreases the efficiency of thermal storage devices [14]. To overcome these challenges, flexible PCMs are developed through methods like encapsulation with elastic shells, incorporating flexible porous structures, and forming polymer networks [15]. Various polymers like unsaturated polyester resin, polymethyl methacrylate, polyvinyl chloride, and thermoplastic elastomer have been used [16,17,18,19,20]. Yang et al. [21] found that using a melamine sponge with excellent elasticity enhanced stability and thermal conductivity. Bing et al. [22] created a UPR/EG/PEG composite that could be used for solar energy applications, and it was found effective in absorbing sunlight, converting photo-thermal energy, and energy storage. However, flexible PCMs that include polymer-supporting structures experience sluggish thermal response due to the low thermal conductivity of organic solid-liquid PCMs.

Researchers have adopted diverse approaches, including utilizing carbon-based materials, metallic fillers, and inorganic ceramic fillers such as MXene, graphite, graphene nanoplatelets (GNPs), carbon nanotubes (CNTs), silver particles, silver nanowires, and spherical alumina (Al_2_O_3_) to enhance the photo-thermal conversion and the thermal conductivity of PCMs [23,24]. For example, Qi et al. [25] constructed a PCM that consisted of graphene foam and PW (i.e., GF/PW), which had a three-dimensional (3D) network, giving it excellent shape stability and a high thermal energy storage density. Notably, it demonstrated a remarkable 87% enhancement in thermal conductivity and an 89% improvement in solar-thermal conversion efficiency. Wei et al. [3] employed a simple physical blending method to integrate GNPs as thermal conductive fillers into a cross-linked polyolefin elastomer (POE), resulting in flexible PCMs with a thermal conductivity of about ∼5.11 W m^−1^K^−1^ and a notable capacity for efficient solar-thermal conversion. Ishida and Rimdusit [26] achieved a thermal conductivity as high as 32.5 W m^−1^K^−1^ in a polybenzoxazine composite containing boron nitride (BN) at a filler concentration of 78.5 vol% (88 wt%). Despite the improved thermal conductivity, the mechanical properties were compromised due to the formation of defects at the polymer–filler interface, which negatively affected the phonon transport and mechanical properties [27]. The observed decline in mechanical properties after filler addition is consistent across various flexible PCMs, posing a challenge to effectively improve thermal conductivity while preserving favorable mechanical properties.

Numerous studies showed that incorporating hybrid fillers improved the performance of functional fillers in polymer composites, enhancing properties like electromagnetic interference shielding [28,29], wave absorption [30,31], electrical conductivity [32], and thermal conductivity [33]. Ren et al. [34] observed enhancements in both mechanical properties and thermal conductivity of polypropylene (PP) through the addition of GNPs. Jin et al. [35] reported an increase in the thermal conductivity of polycarbonate (PC) from 0.19 to 1.42 W m^−1^K^−1^ by adding 20 wt% BN, 1 wt% GNPs, and 1 wt% CNTs. Although significant research progress has been achieved, there remains a lack of comprehensive understanding regarding hybrid fillers in preparing functional polymer composites. Developing PCMs with latent heat and concurrently improving mechanical strength, light-to-heat conversion, and heat transport is necessary to overcome obstacles for experimental design. Further investigation is essential to uncovering the intricate relationship between the structure and performance of PCMs.

In this study, a series of POE/PW/GNPs/Al_2_O_3_ PCMs were prepared using an injection moulding technique. The mechanical strength, flexibility, thermal conductivity, and efficiency in converting light to heat were systematically studied. Additionally, the investigation delved into detailed analyses of morphology, structure, crystallization, thermal stability, and simulated thermal management scenarios. The objective is to develop flexible PCMs with noteworthy thermal conductivity, large latent heat storage, effective light-heat conversion, and high tensile strength. This work aimed to enable integrated functionalities such as photothermal conversion, efficient heat storage, and versatile utilization in various thermal management applications. The proposed method provides a facile method to prepare PCMs with enhanced performance that show promising applications in the energy storage, solar utilization, and advanced thermal management sectors.

## 2. Materials and Methods

### 2.1. Materials

Paraffin wax (PW), with an apparent density of 0.90 g cm^−3^, was obtained from China Sinopharm Group, Beijing, China. Polyolefin elastomer (POE), commercially known as 3980, was provided by ExxonMobil Chemical Co., Ltd., Spring, TX, USA, and it has a density of 0.89 g cm^−3^. Graphene nanoplatelets (GNPs), with a thickness of 4~20 nm and filler size ranging from 5~10 μm, were supplied by Chengdu Institute of Organic Chemistry, Chengdu, China. Spherical alumina (Al_2_O_3_), with an average particle size of 20 μm, was purchased from Tianxing New Materials Technology Co., Ltd., Xiaoyi, China.

### 2.2. Sample Preparation

Prior to blending, POE pellets, Al_2_O_3_, and GNPs were dried at 60 °C for at least 10 h. After this, a sequence of POE/PW/GNPs/Al_2_O_3_ composites were meticulously formulated through melt blending, employing an internal mixer (XSS-300) at 90 °C and 50 rpm for 5 min. The specific formulations for the prepared samples are tabulated in Table 1. Afterwards, the resulting mixtures were pulverized using a grinder, and they were used for injection moulding using a Thermo Scientific HAAKE Minijet apparatus (Waltham, MA, USA). The injection pressure was set at 500 bar, and the volumetric flow rate was about 3.0 cm^3^ s^−1^. The dimensions of the samples were 80 × 10 × 4 mm^3^ (length × width × thickness), and the mold temperature was 40 °C.

### 2.3. Characterization

#### 2.3.1. Differential Scanning Calorimetry (DSC)

To analyze the melting and crystallization behavior of the flexible phase change materials (FPCMs), a differential scanning calorimeter (DSC, TGA/DSC-1, Mettler Toledo, Zürich, Switzerland) was employed. Samples, weighing 5~8 mg, were heated to 200 °C at 10 °C min^−1^, and they were held at 200 °C for 5 min to eliminate thermal history. Subsequently, samples were cooled down to 40 °C at 10 °C min^−1^. All tests were carried out in a nitrogen (N_2_) atmosphere.

#### 2.3.2. Thermal Conductivity

The thermal diffusivity (α) of samples was determined by a laser flash method using a Discovery DXF-900 instrument (TA Instruments, New Castle, PA, USA). To determine the thermal conductivity (λ) of FPCMs, the following equation was employed: λ = α × C_p_ × *ρ*, where *ρ* is density [36]. Here, α and C_p_ denote the thermal diffusivity and specific heat capacity, respectively.

The bulk density was measured using the water displacement method with the help of an MH-120E apparatus (Matsu Haku, Taiwan, China). The C_p_ was determined by DSC using sapphire as the reference.

#### 2.3.3. Scanning Electron Microscope (SEM)

The morphology of FPCMs was observed using a scanning electron microscope (SEM, Nova Nano 450, FEI, Hillsboro, OR, USA). The samples were cryo-fractured in liquid nitrogen, followed by coating a thin layer of gold to enhance image resolution.

#### 2.3.4. Electrical Conductivity

A high-resistance meter (model TH2684A, Changzhou Tonghui Electronics Co., Ltd., Changzhou, China) was used to evaluate the electrical conductivity (σ) of FPCMs. The testing range of the electrometer ranged from 10 kΩ to 100 TΩ. The values of σ for samples were determined using Equation (1) [37]:

σ = 1/ρ = L/RS (1)

where, ρ is electrical resistivity, L is the distance between the copper electrodes, R is the volume resistance, and S is the cross-sectional area.

#### 2.3.5. Viscoelastic Behavior

To delve into the viscoelastic properties, a dynamic rheometer (MCR302, Anton Paar, Graz, Austria) was employed at a temperature of 90 °C. Analyses were performed using a constant-strain mode with a strain rate of 1%. To investigate the storage modulus (G′), viscous modulus (G″), and complex viscosity (η*) at various frequencies ranging from 100 to 0.01 rad s^−1^, specimens with a diameter of 25 mm and thickness of 1 mm were employed.

#### 2.3.6. X-ray Diffraction (XRD)

The crystalline structure and diffraction characteristics of FPCMs were studied using X-ray diffraction using an X-ray diffractometer (XRD, X’Pert Pro, PANalytical, Almelo, The Netherlands). The diffraction patterns were collected within a 2θ range of 5~60° at 5° min^−1^.

#### 2.3.7. Mechanical Properties

The mechanical properties of FPCMs were assessed using an Instron 4302 universal mechanical tester (Instron Co., Canton, MA, USA). The crosshead speed was 50 mm min^−1^.

#### 2.3.8. Light-to-Energy Conversion and Energy Storage

The energy transformation from light to electricity was accomplished with the assistance of a CELHXUV300 xenon lamp (CEAULIGHT, Beijing, China) and an AM 1.5 filter, along with a CEL-NP2000 optical power meter and a Seebeck thermoelectric device at 25 °C. A Keithley electrometer (2400, Cleveland, OH, USA) was utilized to record real-time voltage data.

#### 2.3.9. Light-to-Temperature Energy Conversion

The energy conversion from light to temperature was conducted using a CELHXUV300 xenon lamp (CEAULIGHT, China) equipped with an AM 1.5 filter, a CEL-NP2000 optical power meter (CEAULIGHT, China), and a real-time temperature detector (TA612A, TASI, Suzhou, China). The samples were positioned within a foam insulation system, and the lamp’s simulated sunshine was radiated directly on the surface of samples. The light intensity was measured and adjusted using an optical power meter (CEL-NP2000, Beijing Zhongguo Jinyuan Science and Technology Co., Ltd., Beijing, China) to ensure accuracy. The calibration procedure allowed for exact control and measurement of light intensity during light-to-temperature energy conversions, providing vital insights into the performance of FPCMs under various lighting circumstances.

## 3. Results and Discussion

### 3.1. Rheological Properties

**Figure 1:**
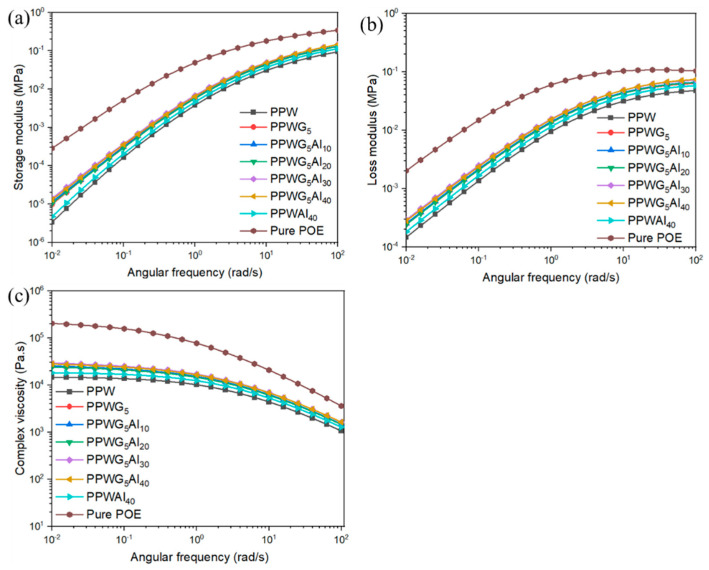
(**a**) Storage modulus (G′), (**b**) loss modulus (G″), and (**c**) complex viscosity (η*) of POE/PW and POE/PW/GNPs/Al_2_O_3_ composites as a function of frequency at 90 °C.

The flowability of composites plays a crucial role in the injection moulding process, which affects the filling properties and microstructural evolution of the products [24,38]. To investigate the effect of adding Al_2_O_3_ and GNPs on the flowability of POE/PW composites, the rheological properties of the composites were tested. Figure 1 presents the frequency-dependent viscoelastic properties of the POE/PW/Al_2_O_3_/GNPs composite, including storage modulus (G′), loss modulus (G″), and complex viscosity (η*). Figure 1a,b indicate that introducing PW into the POE significantly reduces the values of both G′ and G″, revealing that the elasticity of the POE/PW composites is enhanced. However, adding Al_2_O_3_ and GNPs into POE/PW composites displays a reverse phenomenon, in which the G′ and G″ increases with the presence of inorganic fillers. The above revealed that the added fillers had a reinforcing effect on the POE/PW composites. Moreover, the η* of all samples exhibits a gradual decrease with increasing shear frequency within the entire frequency range, known as the “shear thinning” phenomenon [39]. As shown in Figure 1c, all composites exhibit a plateau where the η* of the composites does not change with an increase in a low-frequency region, indicating that the composites possess the characteristics of non-Newtonian fluids. Whereas the η* of the composites decreases with further increasing angular frequency, exhibiting the phenomenon of Newtonian fluid. Nevertheless, the η* for samples containing fillers demonstrates an increase with the incorporation of Al_2_O_3_ and GNPs. The results suggest that the addition of fillers impairs the mobility of polymer melts, causing an increase in η*. It is noteworthy that the η* of the composites is consistently lower than pure POE, indicating enhanced flowability and processability. 

### 3.2. Morphology

**Figure 2:**
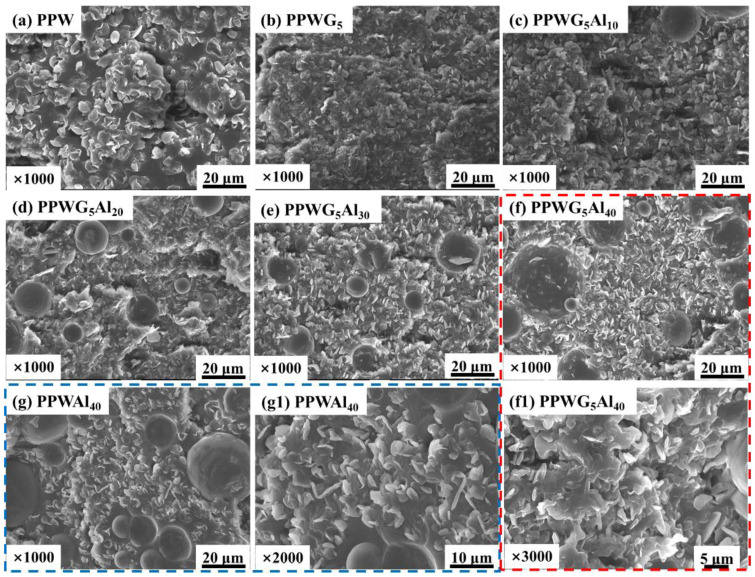
SEM images of FPCMs, in which (**f1**) and (**g1**) were the local magnification of (**f**) and (**g**), respectively.

Figure 2 displays the SEM images illustrating the cross-sectional view of FPCMs. The PW exhibits a dense morphology, and the PW of spherical shapes that are irregular and vary in size are tightly integrated into a composite structure of POE. The irregular spherical shape of PW was attributed to the difference in the wettability between the POE and PW. In Figure 2b, it is evident that the addition of GNPs effectively improves the distribution of PW, resulting in smaller particle sizes. Interestingly, Figure 2c–g show that the presence of Al_2_O_3_ affects neither the morphology nor the structure of PW-based FPCMs. PW is well distributed among the inorganic particles, serving as an effective connector and contributing to enhancing the mechanical properties of the composites.

### 3.3. Crystallization Behavior of FPCMs

**Figure 3:**
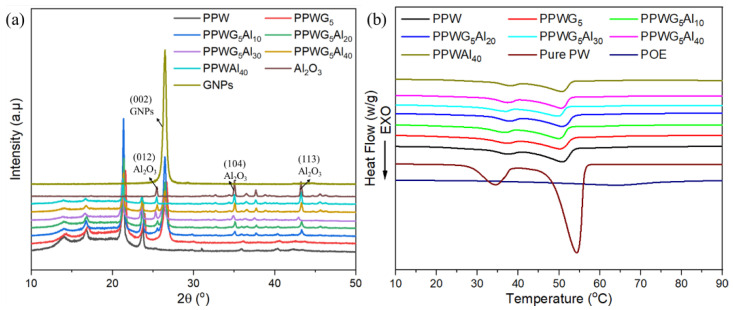
(**a**) The XRD curves and (**b**) DSC curves of FPCMs.

The XRD patterns of FPCMs are shown in Figure 3a. The characteristic peaks of FPCMs are a combination of the peaks of several components (GNPs, Al_2_O_3_, and PW), which proves that the filling process is basically physical mixing. Moreover, compared with pure PPW, the position and intensity of the peaks at 21.4° and 23.8° of FPCMs do not change significantly. This suggests that PW retained its crystalline state within FPCMs, ensuring the effective release of latent heat.

The heat storage capacity, a critical parameter for FPCMs, was determined by DSC. Figure 3b illustrates the DSC curves for FPCMs with varying Al_2_O_3_ loadings. The corresponding results are shown in Table 2. During the melting process, there are two obvious phase transition peaks in the DSC curves of PW, among which, the peak of about 35 °C corresponds to the solid-solid phase transition of PW, and with the further increase in temperature, PW undergoes a solid-liquid phase change; a second endothermic peak appears at about 50 °C, which reflects the good heat storage ability of PW. The DSC curves of FPCMs with different loadings of Al_2_O_3_ and GNPs showed highly overlapping patterns, indicating a similar phase transition behavior, with two endothermal peaks appearing during the melting process. This suggested that the presence of Al_2_O_3_ and GNPs allowed for a normal phase transition process. However, a slight difference between FPCMs and pure PW was noted in the melting temperature. This is mainly because the cross-linking effect of FPCMs resulted in an expansion of PW particles during the melting process.

### 3.4. Mechanical Properties

**Figure 4:**
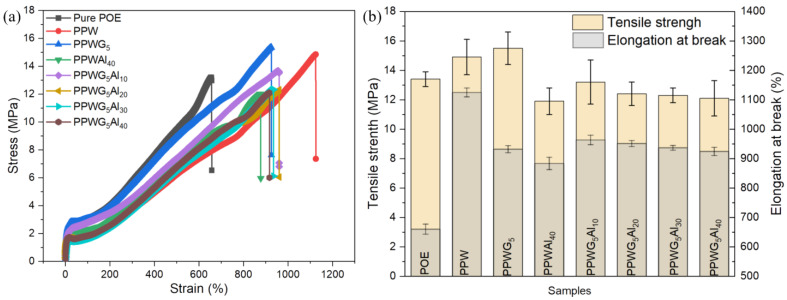
(**a**) Stress–strain curves of FPCMs and (**b**) the corresponding average data.

The application of FPCMs demands commendable mechanical properties, often presenting a challenge since PW has a high degree of rigidity in the solid state. Typically, the simultaneous improvement of mechanical strength and heat storage properties in FPCMs is considered contradictory. Figure 4a illustrates the stress–strain curves of FPCMs filled with varying amounts of Al_2_O_3_, and the results are shown in Figure 4b. Introducing PW into POE proves to be highly effective, significantly enhancing the elongation at break from approximately 650% to around 1100%, representing a twofold improvement. Notably, the PPW, with and without GNPs, demonstrated the ability to withstand tensile stresses of 15.3 and 14.8 MPa, respectively. Correspondingly, the tensile strain reached 930% and 1127%, respectively. The tensile strength and strain of PW-based PCMs demonstrated a regular decrease with increasing Al_2_O_3_ loadings. Even at an Al_2_O_3_ loading of 40 wt%, FPCMs exhibited a tensile stress of 12 MPa and a strain of 900%. FPCMs with mechanical strength serve as an effective thermal management solution. Despite the irregular distribution of Al_2_O_3_ and PW, the cross-linking structures contributed to improving the mechanical strength of FPCMs, enabling a simultaneous enhancement in strong mechanical strength, converting light into heat, and maintaining latent heat.

### 3.5. Thermal Conductivity

**Figure 5:**
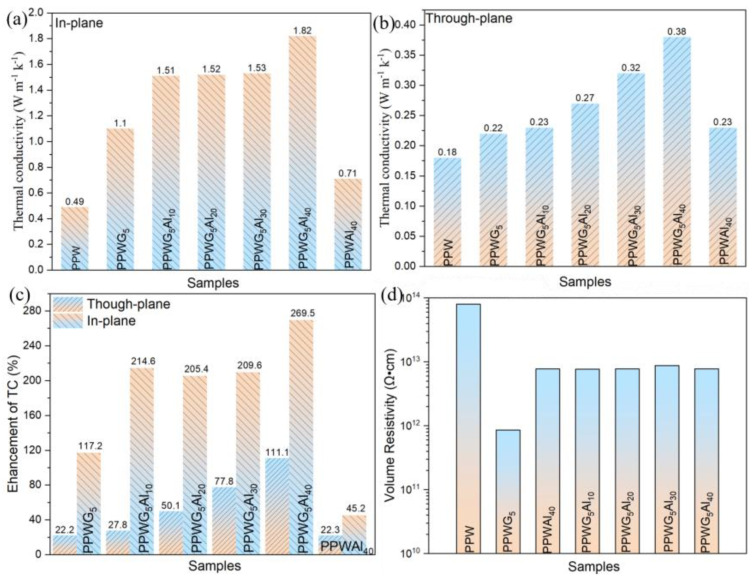
(**a**) In-plane and (**b**) through-plane thermal conductivity (λ) of FPCMs, (**c**) the enhancement of λ for FPCMs when compared with PPW, and (**d**) the volume resistivity of FPCMs.

Figure 5a–c depicts the in-plane and through-plane λ as well as the enhancement rate of λ for POE/PW/Al_2_O_3_/GNPs composites. The λ of PPW without fillers is poor, with in-plane and through-plane λs of 0.49 and 0.18 W m^−1^K^−1^, respectively. The difference in λ between the in-plane and through-plane of PPW is due to the orientation of PPW chains under the action of a shear force field during injection moulding. The λ of PPW increased due to the addition of functional fillers. In particular, the PPWG_5_ had an in-plane λ of 1.1 W m^−1^K^−1^ and a through-plane λ of 0.22 W m^−1^K^−1^. This was related to the oriented arrangement of polymer chains that improved the λ by increasing the mean free path of phonons. Figure 5a,b show that the λ of POE/PW/GNPs/Al_2_O_3_ in different orientations increases linearly with the increase in Al_2_O_3_ content, which suggests that the increase in filler content facilitated the formation of 3D conductive pathways, thereby contributing to an increase in λ. The in-plane and through-plane λs of PPWG_5_Al_40_ are 1.42 and 0.38 W m^−1^K^−1^, which increased by 269% and 111%, respectively, when compared to pure PPW (Figure 5c). This is mainly due to the complementary shapes of Al_2_O_3_ and GNPs, which exert a synergistic effect inside the matrix and significantly improve the λ. Furthermore, the composites have commendable insulating properties, as confirmed by the insulation analysis in Figure 5d. Table 3 shows the λ using similar thermally conductive fillers or phase change materials. It can be noted that this work is the highest in both the values of λ and the degree of increase in λ. This further confirms that the spherical Al_2_O_3_ and lamellar GNPs are able to complement each other to exert a synergistic effect within the matrix, which greatly facilitates the construction of the thermal conductive network.

**Figure 6:**
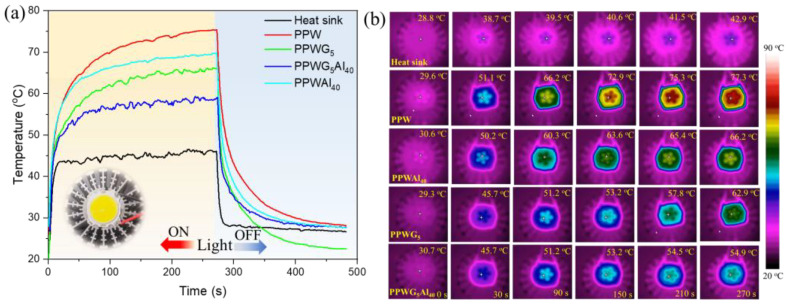
(**a**) The temperature variation of LED with time (the temperature of the center of LED chip was measured) and (**b**) the infrared images of working LED using FPCMs as a thermal management material after 5 min.

The service life of LED lamps decreases exponentially with the increase in operating temperature, and the accumulation of heat causes problems such as wavelength shift and output power reduction. To verify the heat dissipation of thermal management materials, FPCMs with a thickness of 2 mm were placed between the LED chip and heat sink. A temperature recorder and a thermal infrared imager were also used to record the temperature of the LED lamp surface in real time, as shown in Figure 6a,b. The results showed that when using PPW as the thermal management material, the temperature of the LED surface increases gradually with the prolongation of working time, and when the time reaches 270 s, the temperature of the LED surface reaches about 75 °C, since PPW has a lower λ and it is not able to dissipate the heat generated by the LED chip. However, when using POE/PW/GNPs/Al_2_O_3_ composites, the temperature of the LED chip shows a slower increase and stays at a relatively low steady state of 77.3 °C (PPWAl_40_) and 65.2 °C (PPWG_5_), respectively. A comparison of the data reveals that the PPWG_5_Al_40_ has a higher thermal management capability with the concurrent addition of GNPs and Al_2_O_3_. The temperature of the LED chip sustained at 56.9 °C, which is about 13 °C higher than that of heat sink. This fully demonstrates that PPWG_5_Al_40_ has the best heat dissipation ability, which is consistent with its high λ.

### 3.6. Light-to-Heat Conversion

**Figure 7:**
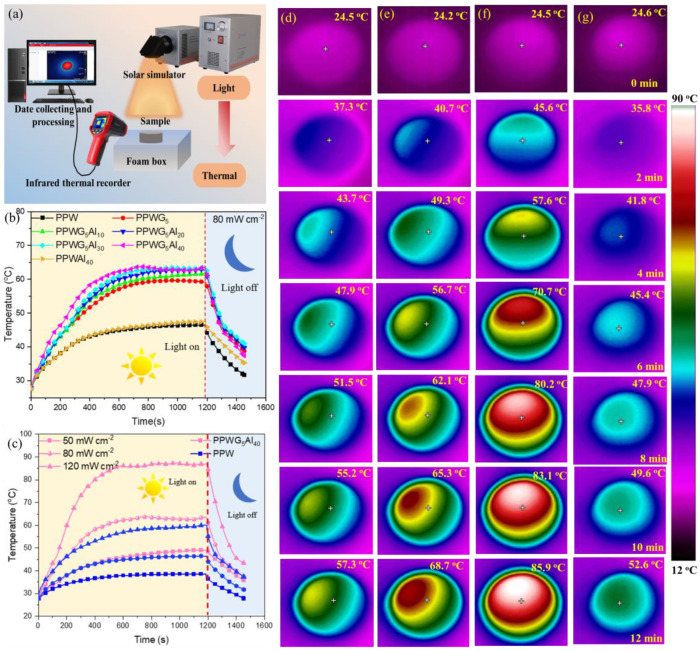
(**a**) Illustration of light-to-thermal energy conversion equipment; (**b**) surface temperatures of FPCMs under various NIR light irradiation; (**c**) surface temperature changes of FPCMs under 80 mW cm^−2^ NIR light irradiation and (**d**–**f**) the infrared thermal images of PPWG_5_Al_40_ composites under 50, 80, and 120 mW cm^−2^ NIR light irradiation, respectively; and (**g**) the infrared thermal images of FPCMs under 120 mW cm^−2^ NIR light irradiation.

As shown in Figure 7a, the ability of photo-thermal conversion was evaluated using a homemade device, in which a composite film was placed on a heat sink, and the change in surface temperature was recorded by an infrared thermographic camera under the simulated sunlight. As shown in Figure 7b, the surface temperature of POE/PW/GNPs/Al_2_O_3_ increases rapidly at a light density of 80 mW cm^−2^, and a temperature plateau appears during the heating process. The maximum temperature of PPW as well as PPWAl_40_ films reaches about 45 °C. However, with the addition of GNPs, the maximum temperature of PPWG_5_ was substantially increased to 60 °C. Moreover, by introducing Al_2_O_3_ into PPWG_5_, the maximum temperature of PPWG_5_Al_y_ can be further enhanced. For example, the PPWG_5_Al_40_ film shows the fastest heating rate with the highest temperature. Therefore, it can be concluded that GNPs and Al_2_O_3_ play a dominant role in photothermal conversion ability. As shown in Figure 7c, the surface temperatures of FPCMs were analyzed at the light intensities of 50, 80, and 120 mW cm^−2^, respectively, and the surface temperatures of FPCMs increased with the increase in light power. PPWG_5_Al_40_ reaches a very high temperature of about 87 °C at a light intensity of 120 mW cm^−2^.

To further validate the impact of GNPs and Al_2_O_3_ on light-to-thermal conversion performance, an analysis of the surface temperature distribution was conducted using an infrared thermal camera for FPCMs. As depicted in Figure 7d, with an increase in irradiation time, the surface temperature of FPCMs consistently increased. Notably, FPCMs with GNPs and Al_2_O_3_ exhibited higher surface temperatures when compared to those without fillers at equivalent irradiation times. Furthermore, a direct correlation was observed between higher sunlight irradiation and the increased surface temperature of FPCMs. For instance, after a 12 min irradiation period, the average surface temperature of PPWG_5_Al_40_ at 50, 80, and 120 mW cm^−2^ reached 57.3, 68.7, and 85.9 °C, respectively. In contrast, the average surface temperature of pure PPW at 120 mW cm^−2^ was only 52.6 °C. These findings further substantiated that the incorporation of GNPs and Al_2_O_3_ effectively enhances the light-to-thermal conversion performance of FPCMs.

**Figure 8:**
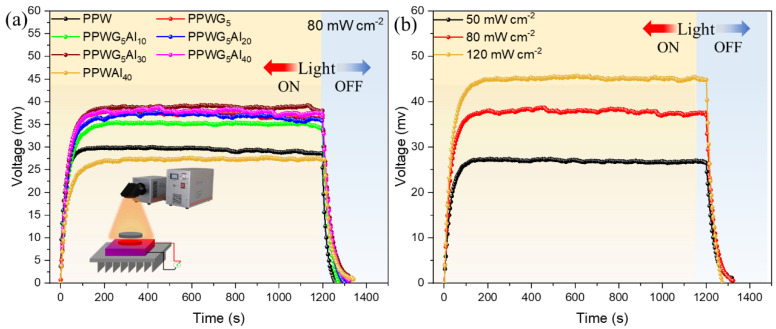
(**a**) Light-to-electric conversion and V-t curves of FPCMs under the sunlight irradiation of 80 mW cm^−2^ and (**b**) V-t curves of PPWG_5_Al_40_ under 50, 80, and 120 mW cm^−2^, respectively.

The GNPs used in this study enhance the photon-absorbing capacity of FPCMs, allowing them to achieve the energy conversion of light property that graphene has demonstrated, which shows considerable promise for light-driven devices [39]. The “Seeback” thermoelectric device was utilized to construct a light-heat-electricity energy conversion that utilizes PCMs. This system utilizes a “Seeback” thermoelectric device, with a composite PCM and tap water serving as the heat source and cold source for the thermoelectric component, as depicted in Figure 8a. The intensity of the simulated sunshine was precisely regulated to 80 mW cm^−2^, while a real-time electrostatic meter was employed to measure the voltage. As shown in Figure 8a, the voltage of POE/PW-based composites increases rapidly at a light intensity of 80 mW cm^−2^, and a clear voltage plateau occurs during the heating process. The maximum voltage of PPW as well as PPWAl_40_ reaches about 26 and 30 mV, respectively. However, with the addition of GNPs, the maximum voltages of PPWG_5_ and PPWG_5_Al_y_ are substantially increased to about 40 mV. Therefore, it can be concluded that the addition of GNPs significantly improves the photo thermoelectric conversion efficiency. As shown in Figure 8b, the photo thermoelectric conversion efficiency of PPWG_5_Al_40_, which has the best photo thermoelectric conversion efficiency, is further investigated by analyzing the voltage of the films at light intensities of 50, 80, and 120 mW cm^−2^, respectively, and the voltage of the films increases with the increase in light power. The PPWG_5_Al_40_ achieves a higher voltage of around 45 mV at a light intensity of 120 mW cm^−2^. It further shows that the photothermal power conversion efficiency of the composite film is better. It is worth mentioning that this work uses tap water as a cold source, and it is close to the actual application scenario.

### 3.7. Thermal Cycling Stability of FPCMs

**Figure 9:**
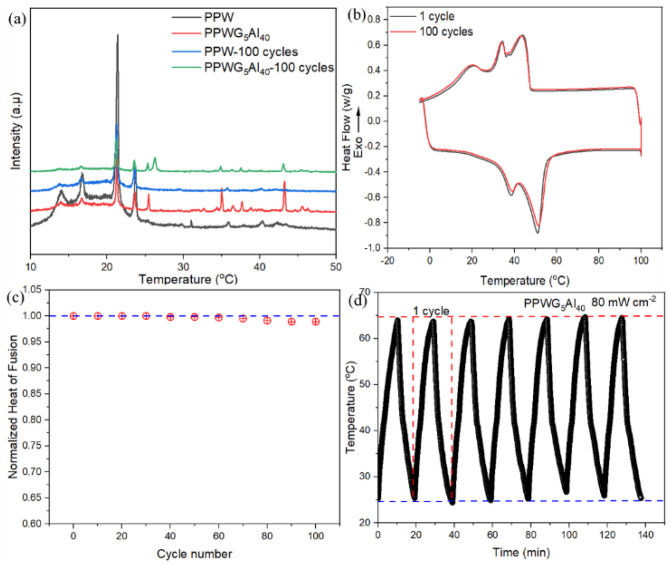
(**a**) The XRD patterns of FPCMs and (**b**) the DSC curves of PPWG_5_Al_40_ with 100 thermal cycles, (**c**) the mass specific heat of fusion normalized by the first heating cycle, and (**d**) the surface temperature changes of FPCMs under 80 mW cm^−2^ NIR light irradiation.

The PPW and PPWG_5_Al_40_ were chosen to analyze the changes of crystalline structures undergoing 100 thermal cycles, as shown in Figure 9a. As can be seen from Figure 9a, no new diffraction peaks appeared in the XRD patterns of all samples before and after cycling, indicating that no change in the crystalline structure of the composites occurred. Moreover, the thermal cycling stability of PPWG_5_Al_40_ was characterized by DSC, as shown in Figure 9b. From the DSC curves in Figure 9b, it can be seen that the phase transition temperature and enthalpy of PPWG_5_Al_40_ did not change significantly after 100 DSC cycles, especially the enthalpy of the phase change basically did not have any loss, which indicates that PPWG_5_Al_40_ can still maintain stable thermophysical properties and chemical structure after 100 cycles of elevated/lowered temperatures, and it exhibits excellent cycling stability. To further validate its stability, multiple cyclic tests were performed on the FPCMs under 80 mW cm^−2^ NIR light irradiation, as displayed in Figure 9d. As can be seen from Figure 9d, the photothermal curves remained unchanged after multiple cycles, indicating that the PPWG_5_Al_40_ exhibits good stability. 

## 4. Conclusions

This work successfully prepared a series of thermal-induced flexible phase change composites (FPCMs) consisting of polyolefin elastomer (POE), paraffin wax (PW), graphene nanoplatelets (GNPs), and aluminum oxide (Al_2_O_3_) through blending and injection moulding. The composites exhibited good thermal management, coupled with superior performance in terms of photothermal conversion and heat transfer. A comprehensive analysis was conducted concerning the synergistic contribution of PW and GNPs to enhance the mechanical strength, the conversion of light to heat, and the heat storage capabilities of FPCMs. The underlying mechanism driving this enhancement was thoroughly elucidated. GNPs play a crucial role as photon-absorbing and molecular heaters that are effective under solar irradiation, inducing lattice vibrations in FPCMs, and facilitating efficient light-to-heat conversion. Furthermore, the combination of GNPs and Al_2_O_3_ established thermal conduction networks, thereby augmenting the heat transfer ability. The synergistic effect of hybrid fillers resulted in the fabrication of PPWG_5_Al_40_ with a tensile strength of 13 MPa, elongation at break of about 900%, in-plane thermal conductivity of 1.82 W m^−1^K^−1^, and efficient improvement in light-to-thermal conversion. In addition to exhibiting favorable crystallization properties, FPCMs displayed high light-driven shape recovery capabilities, acceptable thermal stability, and significant temperature control properties. Collectively, these attributes underscore the suitability of these composites for thermal management applications in electronics, among others.

## Figures and Tables

**Table 1:** Formulations of flexible PCMs.

Designation	Composition			
	POE (wt%)	PW (wt%)	GNPs (wt%)	Al_2_O_3_ (wt%)
PPW	70	30	0	0%
PPWG_5_	70	30	5	0
PPWG_5_Al_10_	70	30	5	10
PPWG_5_Al_20_	70	30	5	20
PPWG_5_Al_30_	70	30	5	30
PPWG_5_Al_40_	70	30	5	40
PPWG_5_Al_40_	70	30	0	40

**Table 2:** The thermal analysis data of DSC.

Samples	Melting Temperature (◦C)	Melting Enthalpy (J/g)	Theoretical Value (J/g)
PPW	50.7	64.9	66.4
PPWG_5_	50.1	48.7	49.1
PPWG_5_Al_10_	49.9	53.4	57.7
PPWG_5_Al_20_	50.6	52.8	53.1
PPWG_5_Al_30_	49.9	64.6	66.4
PPWG_5_Al_40_	50.3	48.3	49.1
PPWAl_40_	50.5	47.4	49.4
PW	53.7	221.2	/

**Table 3:** Comparison of thermal conductivity and enhancement rate of thermal conductivity.

Filler Type	Phase Change Material	Thermal Conductivity (Wm^−1^K^−1^)	Thermal Conductivity Enhancement (%)	Reference
PVA/Graphite	PW	0.29	20.8	[40]
Graphene	PW	0.32	18.5	[41]
OBC/GNPs	Palmitic acid	0.79	155.0	[4]
rGO	PW	0.46	84.2	[19]
Graphene	PW	0.41	17.1	[42]
PVA/MXene	PW	0.62	138.5	[43]
POE/PW/Al_2_O_3_/GNPs	PW	1.82	269.5	This work

## Data Availability

Data are contained within the article.
